# *trans*-Cinnamic and Chlorogenic Acids Affect the Secondary Metabolic Profiles and Ergosterol Biosynthesis by *Fusarium culmorum* and *F. graminearum* Sensu Stricto

**DOI:** 10.3390/toxins9070198

**Published:** 2017-06-22

**Authors:** Tomasz Kulik, Kinga Stuper-Szablewska, Katarzyna Bilska, Maciej Buśko, Anna Ostrowska-Kołodziejczak, Dariusz Załuski, Juliusz Perkowski

**Affiliations:** 1 Department of Botany and Nature Protection, University of Warmia and Mazury in Olsztyn, Plac Łódzki 1, Olsztyn 10-727, Poland; katarzyna.bilska@uwm.edu.pl; 2 Department of Chemistry, Poznan University of Life Sciences, Wojska Polskiego 75, 60-637 Poznan, Poland; kstuper@up.poznan.pl (K.S.-S.); mabu@au.poznan.pl (M.B.); ostrowska.anna.maria@gmail.com (A.O.-K.); julperk@au.poznan.pl (J.P.); 3 Department of Plant Breeding and Seed Production, University of Warmia and Mazury in Olsztyn, Plac Łódzki 3, Olsztyn 10-727, Poland; dariusz.zaluski@uwm.edu.pl

**Keywords:** *Fusarium culmorum*, *Fusarium graminearum* sensu stricto, trichothecenes, phenolic acids, *trans*-cinnamic acid, chlorogenic acid, ergosterol

## Abstract

Plant-derived compounds limiting mycotoxin contamination are currently of major interest in food and feed production. However, their potential application requires an evaluation of their effects on fungal secondary metabolism and membrane effects. In this study, different strains of *Fusarium culmorum* and *F. graminearum* sensu stricto were exposed to *trans*-cinnamic and chlorogenic acids on solid YES media. Fusaria produced phenolic acids, whose accumulation was lowered by exogenous phenolic compounds. In addition, fungi reduced exogenous phenolic acids, leading either to their conversion or degradation. *trans*-Cinnamic acid was converted to caffeic and ferulic acids, while chlorogenic acid was degraded to caffeic acid. The latter underwent further degradation to protocatechuic acid. Fungal-derived *trans*-cinnamic acid, as the first intermediate of the shikimate pathway, increased after chlorogenic acid treatment, presumably due to the further inhibition of the conversion of *trans*-cinnamic acid. Exogenous *trans*-cinnamic and chlorogenic acid displayed the inhibition of mycotoxin production by Fusaria, which appeared to be largely dependent on the phenolic compound and its concentration and the assayed strain. Exogenous phenolic acids showed different effects on ergosterol biosynthesis by fungi. It was found that the production of this membrane sterol was stimulated by *trans*-cinnamic acid, while chlorogenic acid negatively impacted ergosterol biosynthesis, suggesting that phenolic acids with stronger antifungal activities may upregulate ergosterol biosynthesis by Fusaria. This paper reports on the production of phenolic acids by Fusaria for the first time.

## 1. Introduction

Fusarium head blight (FHB) of small grain cereals and Fusarium Ear Rot (FER) of maize remain among the most important diseases affecting cereals worldwide. Among the predominating fungi causing both diseases are *Fusarium graminearum* sensu stricto (s.s.) and *F. culmorum* [1,2]. Besides yield losses, they also cause quality decline by contamination of the grain with type B trichothecenes. This group of mycotoxins includes deoxynivalenol (DON) and nivalenol (NIV), and their acetylated derivatives: 3-acetyldeoxynivalenol (3ADON), 15-acetyldeoxynivalenol (15ADON), and 4-acetylnivalenol (4ANIV, syn. fusarenone X), which have been found to differ in their toxicity towards eukaryote cells [3,4].

**Figure 1:**
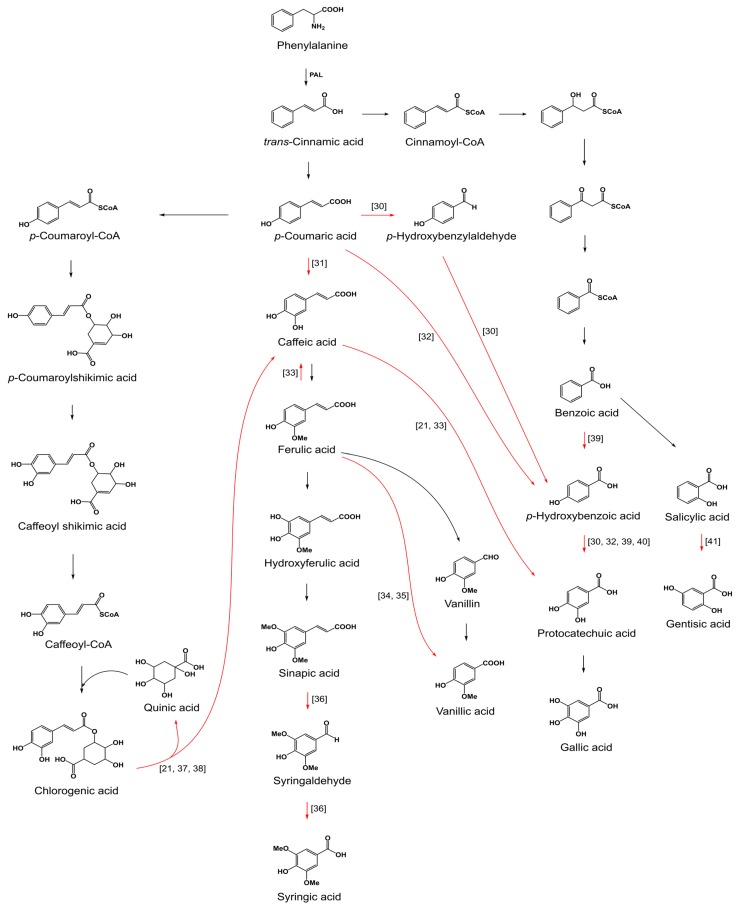
Schematic representation of some of the core biochemical pathways of major phenolic acids (based on Kaushik et al. [30,31,32,33,34,35,36,37,38,39,40,41,42]) with an indication of the routes of the conversion and degradation of phenolic acids by fungi. Red arrows indicate the routes of either the conversion or degradation of phenolic acids by fungi. The numbers near the red arrows indicate the literature from which the data originates.

Currently, fungicide treatment offers the best protection of crops against fungal pathogens, including Fusaria [5]. However, chemical control may not be fully effective in reducing mycotoxin contamination [6], which underlines the continuous need for the development of novel fungicides. Today, however, the need for safer antifungals with a lower environmental impact has become a major public concern [7]. To respond to this demand, a wide range of natural products with a special emphasis on cinnamic-derived phenolic acids have been evaluated for their potential use as alternatives to the current fungicides [7,8,9]. Cinnamic-derived phenolic acids are a group of plant secondary metabolites contributing to disease resistance in plants, mainly as cell wall reinforcers and antifungals [7,10,11,12]. The production of these compounds is regulated by phenylalanine ammonia-lyase (*PAL*), which begins their formation with the transformation of phenylalanine into *trans*-cinnamic acid. This phenolic acid undergoes other enzymatic transformations, yielding a diversity of related phenylpropanoids [13]. Some of the core biochemical pathways of major phenolic acid are depicted in Figure 1. 

The potential of cereal cultivars to induce phenolic acid accumulation appears to play a primary role in restricting fungal infection [14]. For example, Atanasova-Penichon et al. [15] showed that maize genotypes with enhanced resistance against Fusaria can generate close to 2200 μg/g of chlorogenic acid in developing kernels, which is at least 44-fold more than in susceptible genotypes. However, the current interest in phenolic acids arises not only from their antifungal properties, but rather from their potential inhibitory effects on mycotoxin production resulting from their antioxidant and antiradical properties [14]. The latter contribute to their potential of scavenging reactive oxygen species (ROS), which have been found to induce mycotoxin production by Fusaria both in vitro and *in planta* [16,17]. 

The efficient inhibitory activities of phenolic acids towards mycotoxin production were demonstrated against strains of *F. culmorum* and *F. graminearum* s.s. [9,18,19,20]. However, despite a large body of evidence on their protective role against Fusaria, not all studies provide clear evidence on the inhibitory effect of phenolic acids on trichothecene production. For example, caffeic and ferulic acids are known to inhibit mycotoxin production [9,19,21]. However, studies by Ponts et al. [22] and Etzerodt et al. [23] supported opposite results, indicating a stimulative effect on trichothecene biosynthesis. It has been indicated that different in vitro conditions could result in contradicting data [23,24]. Therefore, special attention should be paid to ensure the stability of phenolic compounds in in vitro systems. In addition, another aspect considered in such studies should aim at studying the potential transformation of exogenously applied phenolic acids by fungi [21], especially due to their ability to form phenolic-derived degradation products. Finally, since the discovery of the *PAL* gene in *F. verticillioides* (accession number XP_018747958) [25], further studies are required to confirm the production of phenylpropanoids by fungi. The shikimate pathway is highly conserved, being present in plants, algae, fungi, and even in some prokaryotes [26], although the production of phenolic acids has only been detected in a few fungal species [27,28,29].

The present study reports on the production of phenolic acids by Fusaria for the first time and analyzes whether treatment with *trans*-cinnamic and chlorogenic acids could impact fungal secondary metabolic profiles. The compounds included in the study are formed in different steps of the metabolic route of the shikimate pathway (Figure 1), which was the main criterion based on which they were chosen. It was found that treatment with both *trans*-cinnamic and chlorogenic acids affected changes in fungal phenolic acid profiles, which appeared to be compound-dependent and linked to the ability of Fusaria to either reduce or convert exogenous phenolic acids. 

The doses of phenolic acids used in the in vitro experiments are in the range of chlorogenic acid accumulated in different maize genotypes in the response of fungal infection [15]. An evaluation was also made for the impact of exogenous phenolic acids on both trichothecene accumulation in the media and the expression of *Tri* genes (*Tri4*, *Tri5*, and *Tri10*) responsible for trichothecene biosynthesis. Finally, it was found that exogenous phenolic acids may impact the biosynthesis of ergosterol by either the stimulation or inhibition of the production of this membrane sterol. The results presented in this paper provide new data on the response of Fusaria to phenolic acids, which may have important implications in developing strategies aimed at limiting mycotoxin levels in food and feed.

## 2. Results and Discussion

### 2.1. Production of Phenolic Acids by F. culmorum and F. graminearum s.s.

Chemical analyses of fungal cultures incubated on YES medium (YES + fungal controls) showed an increased accumulation of phenolic acids, compared to the YES-only control (Supplementary File 1). *trans*-Cinnamic acid, as the precursor of the other phenylpropanoids, comprised 7–11% of the phenolic acids produced by Fusaria.

Among the cinnamic-derived acids, ferulic acid was quantified at the highest levels, comprising nearly 21.5–24% of the phenolic acids. *p*-Coumaric acid, which in plants gets converted into ferulic acid by the hydroxylation and methylation reaction [43,44], was quantified at slightly lower levels than ferulic acid, comprising 19–22.5% of the phenolic acids. Caffeic acid, as an intermediate metabolite between *p*-coumaric and ferulic acid, reached 7–8% of the phenolic compounds. Fungal cultures accumulated considerable amounts of sinapic acid, which has been shown to be transformed in plants from ferulic acid [44]. The sinapic acid concentration in fungal cultures yielded 13–15% of the phenolic acids. Fungi also produced syringic acid at levels comprising 7% of the phenolic acids, presumably due to the transformation of sinapic to syringic acid, which appears to be common in filamentous fungi [36]. Among the cinnamic-derived acids, chlorogenic acid was quantified at the lowest levels, comprising nearly 5–6% of the phenolic acids.

Besides cinnamic-derived acids, Fusaria produced benzoic acids, among which gallic acid accumulated at the highest levels, comprising 5–8% of the phenolic acids. Other benzoic acid derivatives, such as gentisic and *p*-hydroxybenzoic acids, were quantified at much lower amounts, yielding 2–4% of the phenolic acids. Among the benzoic acids, protocatechuic acid was produced at the lowest levels, yielding 0.2–0.4% of the phenolic acids.

The biosynthesis of phenylpropanoids is encoded by the *PAL* gene family, which has been well-characterized in plants, including angiosperms and gymnosperms [45]. The protein sequence of phenylalanine ammonia-lyase from *F. verticillioides* can be found in the GenBank database under accession number XP_018747958 [25]. The conducted BLASTP search of its 723-amino acid sequence showed a high (83–95%) identity to hypothetical proteins from the lyase I-class-like superfamily containing histidine ammonia-lyase and phenylalanine ammonia-lyase in the range of *Fusarium* species including *F. graminearum* s.s. (100% query cover, *E* value 0.0). Thus, it can be predicted that the production of phenylpropanoids is common in the genus *Fusarium*, which should receive considerable attention in the future, especially due to the prominent and diverse roles they play, especially with respect to stress protection and cellular signaling [45,46]. 

### 2.2. Exogenously Applied trans-Cinnamic and Chlorogenic Acids Contribute to Changes in the Production of Phenolic Acids in Fungal Cultures of F. culmorum and F. graminearum s.s.

Yeast extract-sucrose (YES) agar medium was supplemented with 100, 400, and 800 μg/g of *trans*-cinnamic and chlorogenic acids to study the effect of these compounds on phenolic acid production by fungi. These concentrations were selected to correspond to <25%, <100%, and <200% of the sum of the phenolic acids produced by fungi, respectively. In both YES + *trans*-cinnamic acid and YES + chlorogenic acid controls, the amount of (initially dissolved in ethanol) phenolic acid was unaffected after the incubation period (Supplementary File 1). This can be considered an advantage over liquid medium where chlorogenic acid, as previously shown, undergoes isomerization [21,47]. Other tested phenolic acids were present in phenolic-acid-treated controls at levels close to that found in the YES-only control. 

Fungi exposed to either 100 or 400 μg/g *trans*-cinnamic acid reduced this phenolic compound by 88–90% and 85–88%, respectively. The conversion of a higher concentration (400 μg/g) of *trans*-cinnamic acid led to the formation of caffeic and ferulic acid, whose concentrations increased by 4–5 and 1.5–2-fold, respectively, as compared to the YES + fungal controls. 

All other phenolic acids, as well as the sum of the phenolic acids, decreased after treatment with either 100 or 400 μg/g *trans*-cinnamic acid, which suggests an inhibition of *PAL* activity by exogenous *trans*-cinnamic acid. However, it is not clear why the conversion of exogenously applied *trans*-cinnamic acid was only limited to the formation of caffeic and ferulic acid—the first intermediates of the shikimate pathway. A possible explanation may be that the incubation period of fungi was too short for their further transformation.

Fungi exposed to either 100, 400, or 800 μg/g chlorogenic acid reduced this phenolic compound by 50–58%, 65–69%, and 58–62%, respectively. Chlorogenic acid can be hydrolyzed into caffeic acid by chlorogenic acid hydrolases [21]. In addition, caffeic acid can be further converted into protocatechuic acid through enzymatic oxidation followed by the decarboxylation of caffeic acid [21,38]. Compared to the YES + fungal controls, caffeic acid increased by 2.5–3 and 3–3.5-fold after treatment with 400 and 800 μg/g chlorogenic acid, respectively. An increase of caffeic acid was not evident in cultures exposed to 100 μg/g chlorogenic acid, probably due to the efficient degradation of caffeic acid to protocatechuic acid, which dramatically increased by 11–24-fold, compared to the YES + fungal controls. A more efficient degradation of chlorogenic acid to protocatechuic acid was evident in fungal cultures exposed to 400 and 800 μg/g of chlorogenic acid. 

Interestingly, a dramatic accumulation of *trans*-cinnamic acid was identified, which increased by 1–1.5, 4–6.5, and 6–9-fold after treatment with 100, 400, and 800 μg/g chlorogenic acid, respectively. Other *trans*-cinnamic acid derivatives (ferulic, *p*-coumaric, sinapic, syringic, gallic, gentisic, and *p*-hydroxybenzoic acid) decreased, which may suggest suppression in converting the *trans*-cinnamic acid further. It is hypothesized that this dramatic increase of *trans*-cinnamic acid might be linked to the over-accumulation of caffeic acid, which was not efficiently converted to the other phenolic acids due to its late formation. Importantly, the sum of phenolic acids was significantly lower in chlorogenic-acid-treated cultures than in corresponding YES + fungal controls, which indicates the inhibition of *PAL* expression by chlorogenic acid. 

### 2.3. Exogenous trans-Cinnamic and Chlorogenic Acids Lowers Trichothecene Accumulation in the Media

The fungal strains used in this study co-produced trichothecene compounds in different amounts. DON chemotypes co-produced DON and acetyl derivatives of DON, while NIV and/or 4ANIV were produced by NIV chemotypes (Table 1). The majority of tested strains co-produced trichothecenes that were not characteristic for their chemotypes in slight amounts, which is typical when solid media is used [48,49] (data not shown). 

The effect of both *trans*-cinnamic and chlorogenic acids on mycotoxin accumulation appears to be largely dependent on the phenolic compound and its concentration and the assayed strain (Table 1). 

Fungi kept at 0.7 mM (100 μg/g) *trans*-cinnamic acid displayed a reduction in mycotoxin content of 15–95%. Higher doses of *trans*-cinnamic acid (2.7 mM, 400 μg/g) reduced mycotoxin levels by 30–95%. The results of the current study support the previous study of Gauthier et al. [21], showing that mycotoxin accumulation in the media can either be increased or decreased by chlorogenic acid, which underlines the risk associated with the enhancement of mycotoxin production by low levels of chlorogenic acid. Four of six strains showed a 23–93% decrease of the sum of mycotoxin accumulation at 0.3 mM (100 μg/g) chlorogenic acid. Higher levels of chlorogenic acid (1.1 mM, 400 μg/g) in the media caused a decrease in mycotoxin content in all six fungal cultures by 20–89%. Similarly, all tested strains incubated in 2.3 mM (800 μg/g) of chlorogenic acid showed a reduction of mycotoxin accumulation by 32–89%.

### 2.4. Gene Expression Studies Reveal Stronger Inhibitory Effect of trans-Cinnamic than Chlorogenic Acid on the Activity of Tri Genes Involved in Trichothecene Biosynthesis 

The effect of both *trans*-cinnamic and chlorogenic acids on gene expression was evaluated by the quantification of *Tri* transcripts of *Tri4*, *Tri5*, and *Tri10* genes on day three of incubation (Table 1). The highest abundance of transcripts was observed for *Tri4*, followed by *Tri5* and *Tri10* genes for all examined strains (data not shown), which is in agreement with the previously examined expression patterns of *Tri* genes [50,51,52]. *Tri5* encodes a terpene cyclase which converts farnesyl pyrophosphate to trichodiene, the hydrocarbon precursor of trichothecenes. Trichodiene is oxygenated by cytochrome P450 monooxygenase encoded by *Tri4*. The expression of both of them is regulated by *Tri10* [52,53]. *trans*-Cinnamic acid showed a more efficient inhibition of *Tri* gene activity than chlorogenic acid. The decrease in *Tri* transcript levels was revealed in most *trans*-cinnamic acid treated cultures, with the exception of *F. graminearum* s.s. strains, whose fold-change values (*P*(H1) = 0.001) in *trans*-cinnamic acid treated cultures (0.7 mM, 100 μg/g) were not significant. In general, the higher dose of *trans*-cinnamic acid assayed, the greater the decrease in *Tri* transcript levels. The decrease in *Tri* transcript levels was revealed in only two cultures (CBS 173.31 and CBS 119173) treated with 1.1 mM (400 μg/g) and 2.3 mM (800 μg/g) chlorogenic acid. The fold-change values (*P*(H1) = 0.001) for the remaining chlorogenic acid treated samples were not significantly altered. 

The stronger inhibitory effect of *trans*-cinnamic than chlorogenic acid on the activity of *Tri* genes may be linked to the increased antioxidant and antiradical properties of *trans*-cinnamic acid (Table 2). The antioxidant and antiradical activities of phenolic acids have been suggested to play a key role in inhibiting trichothecene production by Fusaria [14].

### 2.5. trans-Cinnamic and Chlorogenic Acids Exhibit Different Effects on Ergosterol Biosynthesis by F. culmorum and F. graminearum s.s.

Ergosterol is the predominant component of the fungal cell membrane that is often used to estimate the fungal biomass in different environmental samples [55]. It regulates membrane fluidity and plasma membrane biogenesis and function [56], although no studies on the effect of phenolic acids on ergosterol biosynthesis have yet been conducted. 

Supplementary File 1 shows the effect of both *trans*-cinnamic and chlorogenic acids on the biosynthesis of ergosterol by Fusaria. It was found that the production of this membrane sterol was stimulated in *trans*-cinnamic acid treated cultures, while chlorogenic acid negatively impacted ergosterol biosynthesis. Changes in ergosterol biosynthesis might be explained by the different antifungal activities of phenolic acids. *trans*-Cinnamic acid exhibits remarkably stronger antifungal activity than chlorogenic acid, which, as demonstrated by Ponts et al. [22], can be attributed to the differences in their lipophilicity, scored using retention times (Table 2). The different effects of phenolic acids might also arise from their molecule specific mechanisms of action towards membrane integrity, although there is no data available to support this hypothesis. 

## 3. Conclusions

Fusaria produce phenolic acids. Treatment with either *trans*-cinnamic or chlorogenic acid inhibits the production of phenolic acids and trichothecenes by fungi. Fungal strains reduce exogenous phenolic acids, leading to either their conversion or degradation. Exogenous *trans*-cinnamic acid can be converted to caffeic and ferulic acids. Chlorogenic acids can be degraded to caffeic acid and protocatechuic acids. Fungal-derived *trans*-cinnamic acid, as the first intermediate of the shikimate pathway, increases after chlorogenic acid treatment, presumably due to the inhibition of the further conversion of *trans*-cinnamic acid. Exogenous *trans*-cinnamic and chlorogenic acid display the inhibition of mycotoxin production by Fusaria, which appears to be largely dependent on the phenolic compound and its concentration and the assayed strain. The production of ergosterol is stimulated by *trans*-cinnamic acid, while chlorogenic acid negatively impacts ergosterol biosynthesis. Changes in ergosterol biosynthesis might be explained by the different antifungal activities of phenolic acids.

## 4. Materials and methods

### 4.1. Fungal Strains

Six fungal strains were used in this study (Table 3). They have been maintained in the international fungal collections: Westerdijk Fungal Biodiversity Institute, Utrecht, the Netherlands; MUCL—MUCL Mycothèque de l’Université catholique de Louvain, Louvain-la-Neuve, Belgium; and ARS Culture Collection, USDA, Peoria, IL, US. The detailed description of the fungal strains is given in the ToxGen database [49].

### 4.2. Medium and Culture Conditions

Yeast extract-sucrose (YES) agar medium recommended for trichothecene analysis [57] was used in this study. *trans*-Cinnamic acid and chlorogenic acids (Sigma-Aldrich, Saint Louis, MO, USA) were dissolved in 10 mL of 96% ethanol and then added to YES medium to obtain the final concentrations: 100, 400, and 800 μg/g. The calculated molar concentrations of *trans*-cinnamic acid used in the experiments were: 0.7, 2.7, and 5.4 mM, respectively. For chlorogenic acid, the molar concentrations used were: 0.3, 1.1, and 2.3 mM, respectively. 

This study incorporated three different controls: a YES-only control (YES medium only), YES + *trans*-cinnamic acid and YES + chlorogenic acid controls (YES media supplemented with either 100, 400 or 800 μg/g of phenolic acids), and six YES + fungal controls (fungal strains incubated on YES media). 

Petri plates (Ø 80 mm) were inoculated at the center with a 5-mm agar disc from 6–8-week-old laboratory stock cultures maintained at 4 °C on PDA slants and incubated at 25 °C (in triplicate) in the dark. The current study included chemical and gene expression analyses. Chemical analyses incorporated a determination of phenolic acids and trichothecenes in the media. For gene expression analysis, plates were incubated for three days for each condition, while for chemical analyses, the plates were incubated for 21 days. Strains incubated at the highest concentration of *trans*-cinnamic acid (800 μg/g) were not subjected to any further analysis due to the complete inhibition of fungal growth. 

### 4.3. Determination of Phenolic Acids in the Medium

To reveal the production of phenolic acids by fungi, different phenolic compounds were determined in dried fungal cultures after a 21-day incubation period (Supplementary File 1). Fungal biomass (0.2 g) was placed in sealed 17 mL culture test tubes, where alkaline and acid hydrolysis was conducted. For the alkaline hydrolysis, 1 mL distilled water and 4 mL 2 M aqueous sodium hydroxide was added to the test tubes. Tightly-sealed test tubes were heated in a water bath at 95 °C for 30 min. After cooling (approx. 20 min), the test tubes were neutralized with 2 mL 6 M aqueous hydrochloric acid solution (pH = 2). The samples were then cooled in water with ice. Phenolic acids were extracted from the inorganic phase using diethyl ether (2 × 2 mL). The formed ether extracts were continuously transferred to 8 mL vials and the acid hydrolysis was then conducted. For this purpose, the aqueous phase was supplemented with 3 6 M aqueous hydrochloric acid solution. Tightly-sealed test tubes were heated in a water bath at 95 °C for 30 min. After being cooled in water with ice, the samples were extracted with diethyl ether (2 × 2 mL). The produced ether extracts were continuously transferred to 8 mL vials, after which they were evaporated to dryness in a stream of nitrogen. Prior to analyses, the samples were dissolved in 1 mL methanol. An analysis was performed using an ultra-performance liquid chromatograph (Acquity, Waters, Milford MA, USA) with a Waters PDA Detector.

Chromatographic separation was performed on an Acquity UPLC Shield RP 18 1.7 um 2.1 × 150 mm, with an acetonitrile 0.1% formic acid: 0.1% aqueous formic acid mixture used as an elution phase (gradient). The concentrations of phenolic acids were determined using an internal standard at wavelengths λ = 320 nm and 280 nm. Compounds were identified based on a comparison of the retention time of the analyzed peak with the retention time of the standard, and by adding a specific amount of the standard to the analyzed samples and a repeated analysis. The detection level was 0.1 μg/g. The retention times of the assayed acids were as follows: 52.40 min (*trans*-cinnamic acid), 48.0 (sinapic acid), 46.20 min (ferulic acid), 40.20 min (*p*-coumaric acid), 28.05 min (syringic acid), 26.19 min (caffeic acid), 20.39 min (gentisic acid), 19.46 min (*p*-hydroxybenzoic acid), 14.23 min (chlorogenic acid), 11.36 min (protocatechuic acid), and 8.85 min (gallic acid). Three biological replications were prepared for each condition. 

### 4.4. Determination of Antioxidant Capacity (VCEAC/L) and Radical Scavenging Activity (ABTS) of trans-Cinnamic and Chlorogenic Acids

VCEAC/L and ABTS assays of phenolic acids were performed as previously described by Kim et al. [58] and Re et al. [59], respectively. 

### 4.5. Analysis of Trichothecenes from Fungal Cultures 

The levels of DON, 3ADON, 15ADON, NIV, and 4ANIV were determined in fungal cultures treated and non-treated (YES + fungal controls) with different concentrations of *trans*-cinnamic and chlorogenic acids by GC-MS, as previously described by Perkowski et al. [60] and Kulik et al. [61,62]. Three biological replications were prepared for each condition.

### 4.6. Extraction of total RNA and preparation of cDNA

The total RNA was extracted from three-day-old fungal cultures from mycelium grown on YES medium treated and non-treated (YES + fungal controls) with *trans*-cinnamic and chlorogenic acids. Five biological replications were prepared for each condition. The extraction of RNA and reverse-transcription were performed as previously described in Kulik et al. [61,62]. cDNA samples were stored at −25 °C for transcript quantification.

### 4.7. RT-qPCR and Data Analyses

Two genes (*Tri4* and *Tri5*) responsible for the first steps in the trichothecene biosynthetic pathway were selected for RT-qPCR analysis, as previously described in Kulik et al. [61,62]. The *Tri5* gene encodes trichodiene synthase, a terpene cyclase which converts farnesyl pyrophosphate to trichodiene, the hydrocarbon precursor of trichothecenes [53]. Trichodiene undergoes a series of oxygenations catalyzed by a cytochrome P450 monooxygenase encoded by the *Tri4* gene. The molecular analysis also included the *Tri10* gene responsible for the regulation of multiple *Tri* genes [50]. 

To design primer/probe sets for the expression of *Tri10*, the *F. culmorum* and *F. graminearum* sequences of the *Tri10* gene published in the NCBI database (accession numbers: KU572424 and KU572428) were aligned with Geneious v.6.1.6 (Biomatters Ltd., Auckland, New Zealand, 2014) [63]. 

Due to high polymorphism within the exon-exon boundaries of the *Tri10* gene, two *Tri10* reverse primers were designed for use in separate reactions: *Tri10rc* GCC AAT CTC CCC TGC TTA GA (specific for *F. culmorum*) and *Tri10rg* GCC AAT CTC CCC TGC TTA GG (specific for *F. graminearum*). Forward primer: *Tri10f* CCC TTG CTT GCA TGC TAC AG targeted a conserved binding site. *Tri10* primers and *Tri10* probe (FAM-CAG TTT TGA GTC TTC G-MGB) were designed using PRIMER EXPRESS 3.0 (Applied Biosystems, Foster City, CA, USA). Probes, conjugated with an MGB group, were labeled at the 5′-end with FAM, while the *Ef1α* probe was labeled at the 5′-end with VIC. Primers were synthesized by Sigma-Aldrich (Saint Louis, MO, USA), while MGB probes were ordered from Life Technologies Oligos, Primers, Probes, and Nucleotides Synthesis Service. Duplex RT-qPCR reaction conditions were used for each *Tri* transcript, including the *Ef1α* reference control, as previously described in Kulik et al. [61,62]. The amplification efficiency of the *Tri10* assay was determined based on five five-fold dilutions of the cDNA template of the strain CBS 119173. The amplification efficiency of the *Tri10* assay was 103% with R2 = 0.993 and Slope = 3235, similar to the amplification efficiencies obtained for the remaining assays of Kulik et al. [61,62]. In this study, the relative quantification of *Tri* targets was normalized to an *Ef1α* reference gene. The Ct values of the target *Tri4*, *Tri5*, *Tri10*, and reference *Ef1α* gene were compared in the control and treated samples and normalized relative to the Ct values obtained for the reference *EF1α* gene using the rest 2009 software [64]. 

### 4.8. Determination of Ergosterol 

Ergosterol was quantified in fungal cultures treated with *trans*-cinnamic and chlorogenic acids and in six different YES + fungal controls using UPLC, as described by Perkowski et al. [65]. Three biological replications were prepared for each condition. Ergosterol was also determined in YES-only control and in YES + *trans*-cinnamic and YES + chlorogenic acid controls. 

### 4.9. Statistical Analyses

The significance of the differences among mycotoxin levels was tested using Tukey’s HSD test (*p* < 0.05). The production of phenolic acids was tested by an analysis of the differences among phenolic acid levels between YES-only and YES + fungal controls using non-parametric Kolmogorov-Smirnov and Mann-Whitney U tests at *p* < 0.05. Both non-parametric statistical methods were also used to test the impact of exogenously applied *trans*-cinnamic and chlorogenic acids on the accumulation of other fungal-derived phenolic acids and ergosterol. Differences among phenolic acid/ergosterol levels between YES + fungal controls and samples treated with either *trans*-cinnamic or chlorogenic acid were also tested. The reduction of exogenous *trans*-cinnamic and chlorogenic acids was tested using a *t*-Student test at *p* < 0.05.

## Figures and Tables

**Table 1:** Trichothecene accumulation and RQ (relative quantification) of *Tri* transcripts of fungal strains incubated with different concentrations of *trans*-cinnamic and chlorogenic acids after a 21-day incubation period.

Phenolic Acid Concentration	Strain	Tri Genotype	Trichothecene Levels (mg/kg) (*n* = 3 in Each Condition)						RQ (*n* = 6 in Each Condition)		
			DON	3ADON	15ADON	NIV	4ANIV	Sum of Trichothecenes	*Tri4*	*Tri5*	*Tri10*
YES + fungal controls	MUCL 53469	3ADON	17.5 ± 0.4 (a)	23.1 ± 0.9 (a)				40.6			
	CBS 173.31	3ADON	63.7 ± 3.8 (b)	9.9 ± 0.4 (a)				73.6			
	CBS 139512	NIV				79.2 ± 3.2 (a)	96.4 ± 4.8 (a)	175.6			
	CBS 119173	3ADON	49.6 ± 3.6 (a)	12.2 ± 0.8 (a)				61.8			
	CBS 138561	15ADON	1.5 ± 0.7 (a)		1.5 ± 0.7 (a)			3			
	MUCL 53455	NIV				4.4 ± 0.2 (a)		4.4			
*trans*-cinnamic acid 0.7 mM (100 μg/g)	MUCL 53469	3ADON	4.62 ± 0.14 (c)	16.41 ± 0.33 (b)				21.03	0.21 (0.12–0.38)	0.26 (0.14–0.46)	NS
	CBS 173.31	3ADON	52.48 ± 1.57 (b)	9.9 ± 0.22 (a)				62.38	0.13 (0.11–0.16)	0.19 (0.16–0.23)	NS
	CBS 139512	NIV				68.63 ± 1.37 (b)	15.45 ± 0.62 (b)	84.08	0.14 (0.07–0.28)	0.17 (0.12–0.26)	NS
	CBS 119173	3ADON	2.7 ± 0.2 (b)	0.5 ± 0.03 (b)				3.2	NS	NS	NS
	CBS 138561	15ADON	0.34 ± 0.01 (b)		0.005 ± 0.0002 (b)			0.35	NS	NS	NS
	MUCL 53455	NIV				2.07 ± 0.12 (b)		2.07	NS	NS	NS
*trans*-cinnamic acid 2.7 mM (400 μg/g)	MUCL 53469	3ADON	8 ± 0.2 (b)	17.5 ± 0.5 (b)				25.5	0.02 (0.006–0.038)	0.03 (0.02–0.07)	0.15 (0.1–0.26)
	CBS 173.31	3ADON	44.01 ± 0.88 (c)	7.47 ± 0.37 (c)				51.48	0.02 (0.007–0.055)	0.02 (0.01–0.046)	0.18 (0.1–0.36)
	CBS 139512	NIV				37.26 ± 2.23 (c)	14.12 ± 0.57 (c)	51.38	0.046 (0.044–0.047)	0.023 (0.02–0.03)	NS
	CBS 119173	3ADON	1.8 ± 0.1 (b)	1.3 ± 0.05 (b)				3.1	0.15 (0.089–0.255)	0.21 (0.083–0.541)	NS
	CBS 138561	15ADON	0.22 ± 0.01 (b)		0.003 ± 0.0001 (b)			0.22	0.086 (0.058–0.212)	0.039 (0.028–0.076)	NS
	MUCL 53455	NIV				0.59 ± 0.01 (c)		0.59	0.023 (0.019–0.023)	0.022 (0.02–0.024)	NS
chlorogenic acid 0.3 mM(100 μg/g)	MUCL 53469	3ADON	9.62 ± 0.57 (b)	21.51 ± 0.43 (ab)				31.13	NS	NS	NS
	CBS 173.31	3ADON	84.13 ± 3.37 (a)	11.52 ± 0.46 (a)				95.65	NS	NS	NS
	CBS 139512	NIV				32.3 ± 1.9 (d)	10.3 ± 0.4 (c)	42.6	NS	NS	NS
	CBS 119173	3ADON	3.2 ± 0.2 (b)	1.0 ± 0.1 (b)				4.2	NS	NS	NS
	CBS 138561	15ADON	1.88 ± 0.11 (a)		0.12 ± 0.01 (b)			2	NS	NS	NS
	MUCL 53455	NIV				2.41 ± 0.15 (c)		2.41	NS	NS	NS
chlorogenic acid 1.1 mM (400 μg/g)	MUCL 53469	3ADON	7.93 ± 0.32 (c)	15.66 ± 0.47 (c)				23.59	NS	NS	NS
	CBS 173.31	3ADON	54.88 ± 1.31 (b)	4.27 ± 0.02 (a)				59.15	0.68 (0.65–0.71)	NS	NS
	CBS 139512	NIV				49.6 ± 3 (c)	22.1 ± 1.3 (b)	71.7	NS	NS	NS
	CBS 119173	3ADON	5 ± 0.3 (b)	1.7 ± 0.1 (b)				6.7	0.43 (0,36–0,5)	0.59 (0.55–0.65)	0.89 (0.85–0.93)
	CBS 138561	15ADON	0.29 ± 0,01 (b)		0.07 ± 0.01 (b)			0.36	NS	NS	NS
	MUCL 53455	NIV				3.14 ± 0.1 (b)		3.14	NS	NS	NS
chlorogenic acid 2.3 mM (800 μg/g)	MUCL 53469	3ADON	7.42 ± 0.37 (c)	20.3 ± 0.81 (b)				27.72	NS	NS	NS
	CBS 173.31	3ADON	41.97 ± 1.68 (c)	2.6 ± 0.16 (b)				44.57	0.05 (0.04–0.05)	0.12 (0.11–0.14)	0.17 (0.15–0.2)
	CBS 139512	NIV				57 ± 1.1 (b)	16.5 ± 0.8 (bc)	73.5	NS	NS	NS
	CBS 119173	3ADON	4.9 ± 0.3 (b)	1.8 ± 0.1 (b)				6.7	0.47 (0.45–0.49)	0.71 (0.65–0.71)	0.82 (0.77–0.86)
	CBS 138561	15ADON	1.16 ± 0.07 (ab)		0.05 ± 0.002 (b)			1.21	NS	NS	NS
	MUCL 53455	NIV				1.85 ± 0.1 (d)		1.85	NS	NS	NS

Degree of inhibition: <25%; 25–50%; 50–75%; >75%. Increase of mycotoxin accumulation. (a), (b), (c) letters indicate homogenous groups at *p* < 0.05 followed by the Tukey test. NS—not significant.

**Table 2:** Antioxidant capacity (VCEAC/L), radical scavenging activity (ABTS), and retention times (min) of the *trans*-cinnamic and chlorogenic acids analyzed in this study.

Phenolic Acid	VCEAC/L	ABTS (μmol TROLOX/100 g s.m.)	Retention Times (min) *
*trans*-Cinnamic acid	812.3	314.9	9.6
Chlorogenic acid	12.3	57.1	2.6

* From Labronici Bertin et al. [54].

**Table 3:** List of fungal isolates used in this study.

Species	Strain	Trichothecene Genotype	Origin, Host and Year of Isolation
*F. culmorum*	CBS 173.31, NRRL 26853	3ADON	Canada, oat, 1927
	MUCL 53469	3ADON	Belgium, corn, 2007
	CBS 139512	NIV	Poland, wheat kernel, 2003
*F. graminearum* s.s.	CBS 119173, NRRL 38369	3ADON	USA, Louisiana, wheat head, 2005
	CBS 138561	15ADON	Poland, wheat kernel, 2010
	MUCL 53455	NIV	Belgium, corn, 2007

## Supplementary Materials

The following are available online at www.mdpi.com/2072-6651/9/7/198/s1, Supplementary file 1: Phenolic acid and ergosterol accumulation after 21 days of incubation.
